# Characterization of a Novel Moderately Thermophilic Solvent-Tolerant Esterase Isolated From a Compost Metagenome Library

**DOI:** 10.3389/fmicb.2019.03069

**Published:** 2020-01-24

**Authors:** Ji-Min Park, Chul-Hyung Kang, Sung-Min Won, Ki-Hoon Oh, Jung-Hoon Yoon

**Affiliations:** ^1^Department of Food Science and Biotechnology, Sungkyunkwan University, Suwon, South Korea; ^2^Green Chemistry and Environmental Biotechnology Program, School of Science, University of Science and Technology, Daejeon, South Korea; ^3^Korea Research Institute of Bioscience and Biotechnology, Daejeon, South Korea

**Keywords:** metagenome, lipolytic enzyme, esterase, family IV, solvent tolerant

## Abstract

A novel esterase, EstCS1, was isolated from a compost metagenomics library. The EstCS1 protein, which consists of 309 amino acid residues with an anticipated molecular mass of 34 kDa, showed high amino acid sequence identities to predicted esterases and alpha/beta hydrolases (59%) from some cultured bacteria and to predicted lipases/esterases from uncultured bacteria. The phylogenetic analysis suggested that the EstCS1 belongs to the hormone-sensitive lipase family of lipolytic enzyme classification and contains a catalytic triad including Ser155–Asp255–His285. The Ser155 residue of the catalytic triad in the EstCS1 was located in the consensus active-site motif, GXSXG. Besides, a conserved HGGG motif placed in an oxyanion hole of the hormone-sensitive lipase family was discovered, too. The EstCS1 demonstrated the highest activity toward *p*-nitrophenyl propionate (C3) and caproate (C6) and was normally stable up to 60°C with optimal activity at 50°C. In addition, an optimal activity was observed at pH 8, and the EstCS1 possessed its stability within the pH range between 5 and 10. Interestingly, EstCS1 had an outstanding stability in up to 30% (v/v) organic solvents and activity over 50% in the presence of 50% (v/v) acetone, ethanol, dimethyl sulfoxide (DMSO), and *N*,*N*-dimethylformamide. The EstCS1 hydrolyzed sterically hindered tertiary alcohol esters of *t*-butyl acetate and linalyl acetate. Considering the properties, such as the moderate thermostability, stability against organic solvents, and activity toward esters of tertiary alcohols, the EstCS1 will be worthwhile to be used for organic synthesis and related industrial applications.

## Introduction

Lipases (EC 3.1.1.3) and esterases (EC 3.1.1.1) are α/β hydrolases that are produced by a lot of microorganisms and eukaryotic organisms (Bornscheuer, 2002). Lipases catalyze the synthetic and hydrolytic reaction of slightly long-chain triacylglycerols and have quite low water solubility, whereas esterases that are partially soluble in water express a preference for the catalysis of short-chain triacylglycerols (Arpigny and Jaeger, 1999; Gupta et al., 2004). Lipolytic enzymes have the catalytic triad formed by Ser–Asp/Glu–His residues, and the Ser residue is generally conserved in the Gly–X–Ser–X–Gly pentapeptide motif (Bornscheuer, 2002). Lipolytic enzymes have drawn significant attention on account of useful and unique catalytic properties such as broad substrate specificity, organic solvent stability, position selectivity, and stereoselectivity, which is worthy of being used in agriculture, pharmaceutical, food, and fine chemical industries (Bornscheuer, 2002; Panda and Gowrishankar, 2005; Tutino et al., 2009).

Bacterial and metagenomic lipolytic enzymes have been classified into 17 families (families I–XVII) according to their biological attributes and amino acid sequence (Arpigny and Jaeger, 1999; Castilla et al., 2017). Among the 17 families showing bacterial lipolytic enzymes, family IV showed noticeable sequence similarity to mammalian hormone-sensitive lipases (HSLs), which play a momentous role in regulation of lipid metabolism (Yeaman et al., 1994; Arpigny and Jaeger, 1999; Kim, 2017). The genetic libraries were obtained directly from environmental sources without microbial culture in a laboratory by means of screening methods based on activity and sequence to effectively identify novel enzymes being used in various industries (Lee et al., 2006; Schmeisser et al., 2007; Kim et al., 2009; Uchiyama and Miyazaki, 2009; Berini et al., 2017). Especially, a number of lipases and esterases belonging to family IV have been recently isolated from various environmental sources since the development of metagenomic analyses (Oh et al., 2012; Choi et al., 2013; Privé et al., 2015; Kim, 2017; Huo et al., 2018; Jayanath et al., 2018; Noby et al., 2018).

Soil composting is a process of biological decomposition and humification of organic materials by microorganisms existing in the environment, and its temperature generally rises up to 80°C during the thermogenics period in the process. Thermophiles belonging to the genera *Thermus*, *Bacillus*, *Hydrogenobacter*, and *Geobacillus* have been commonly isolated from that compost (Beffa et al., 1996; Poli et al., 2006). Hydrolytic enzymes including cellulases, hemicellulases, proteases, phosphatases, arylsulfatases, and lipases are released by those microorganisms and play key roles in the composting process (Goyal et al., 2005). Therefore, the compost may be a favorable habitat to search for enzymes with thermostable characteristics.

While metagenomic genetic libraries were constructed from a compost of South Korea to screen novel thermostable lipolytic enzymes, some clones showing lipolytic activity were isolated. Among the genes subcloned, one lipolytic gene (designated *estCS1*) belonging to the family IV and indicating the strongest activity was chosen for further study. In this study, we describe methods and experimental results of cloning, expression, and biochemical characterization of this novel lipolytic enzyme.

## Materials and Methods

### Strains and Plasmids

*Escherichia coli* strain EPI300-T1R (Epicenter) was used for the construction of a metagenomic library. *E. coli* DH5α (Intron biotechnology) and BL21(DE3) (Invitrogen) were used as host strains for subcloning and overexpression of metagenomic gene retaining lipolytic activity, respectively. The pCC1FOS (Epicenter, United States) and pUC19 vectors were used for subcloning of the open reading frame encoding lipolytic activity. The pET22b(+) vector (Novagen) was lastly used for cloning and overexpression.

### Construction of the Metagenomic Library and Screening of Lipolytic Genes

Metagenomic DNA was extracted from a compost sample as previously described (Zhou et al., 1996). That genetic information was collected for the creation of metagenomic libraries with the CopyControl^TM^ fosmid library production kit (Epicenter) in accordance with the manufacturer’s instructions. For esterase/lipase screening based on enzymatic activities, transformants were incubated on Luria–Bertani (LB) agar media containing 1% (v/v) emulsified tributyrin (C4) as a lipid substrate with chloramphenicol (25 μg/ml) and 0.5% (w/v) gum arabic. Positive colonies forming clear halos on the agar media were singled out as candidates for novel lipolytic enzymes, and those genes were sequenced through a random shotgun sequencing method.

### Sequence Analysis

The open reading frames encoding the esterase/lipase were compared with reference sequences that were retrieved from protein and nucleotide databases in the National Center for Biotechnology Information (NCBI). Sequence similarity was investigated through the Protein BLAST program at the NCBI, and multiple alignments were processed with sequences having high similarity by means of the Clustal W program. The phylogenetic tree was constructed using MEGA X program (Kumar et al., 2018) with 1,000 bootstrap replicates; numbers at branching points indicate the percentage of consensus.

### Cloning and Overexpression of the estCS1 Gene

The putative esterase gene was amplified by PCR using forward and reverse primers (5′-GCGCATATGTCTATTCACCCA-3′ and 5′-GGCCTCGAGCTTTTCACGA ATG-3′; the underlined letters indicate the *Nde*I and *Xho*I recognition sites, respectively). Ligation of the PCR fragment and the pET-22b(+) vector was carried out after they were cleaved with *Nde*I and *Xho*I. The recombinant plasmid DNA, designated pET-estCS1, was transformed to *E. coli* BL21 (DE3). The transformed *E. coli* was incubated in LB broth medium with ampicillin (100 μg/ml) at 37°C. When the optical density of the culture attained an absorbance of ∼0.6 at 600 nm, 0.5 mM isopropyl-D-thiogalactopyranoside was added to induct the protein overexpression, and these *E. coli* cells were additionally incubated for 18 h at 21°C. The cells were harvested by centrifugation and resuspended in binding buffer (50 mM Tris–HCl at pH 8.0 containing 300 mM NaCl). Resuspended cells were then sonicated for disruption, and the crude cell lysate was centrifuged in 16,000 rpm at 4°C for 30 min. The supernatant including the cell extract was loaded on to a selective column packed with nickel–nitrilotriacetic acid (Ni–NTA) resin (QIAGEN GmbH). After washing the adsorbed resin using a buffer containing 2 mM imidazole, the bound proteins having high affinity with a Ni-NTA were slowly eluted with a buffer featuring 250 mM imidazole. Concentration of the eluted fractions was conducted by centrifugation in 5,000 × *g* at 4°C. The concentrated fractions were transferred to a gel filtration column (Superdex 200 10/300 GL; GE Healthcare) equilibrated with 50 mM Tris–HCl buffer (pH 8.0) including 300 mM NaCl and consecutively separated at a flowrate of 0.5 ml/min using the BioLogic DuoFlow Chromatography System (Bio-Rad Laboratories). The target protein fraction showing the sharp peak in the gel filtration chromatography was analyzed through sodium dodecyl sulfate (SDS) polyacrylamide gel electrophoresis) in 12% polyacrylamide gels.

### Biochemical Characterization of EstCS1

The concentration of the enzyme was measured in accordance with the method of Bradford (Bio-Rad Protein Assay) with bovine serum albumin as a standard. The standard assay was performed by blending 10 mM *p*-nitrophenyl butyrate (C4), ethanol, and 100 mM GTA buffer (100 mM 3,3-dimethylglutaric acid, 100 mM Tris–HCl, and 100 mM 2-amino-2-methyl-1,3-propanediol) as a mixture (1:4:95, by vol). During the reaction, the absorbance at 405 nm was continuously measured at regular intervals for quantifying the *p*-nitrophenol released from the *p*-nitrophenyl butyrate in the mixture using an Eon spectrophotometer (BioTek). One unit of enzyme activity was defined as the amount of enzyme required to produce 1 μmol of *p*-nitrophenol per minute at 25°C. The preference for *p*-nitrophenyl esters (pNPEs) as substrates (acetate, C2; propionate, C3; butyrate, C4; caproate, C6; caprylate, C8; caprate, C10; laurate, C12) was evaluated enzymatically through gauging the quantity of *p*-nitrophenol released during hydrolysis. To confirm the optimal reaction temperature, the reaction mixtures were incubated with no shaking for 10 min at varied temperatures (10–80°C). Thermostability of EstCS1 was ensured by preincubation in the 100 mM GTA buffer (pH 9.0) for 1 h under the same temperature conditions as the previous experiment, and then, the residual activity was gauged at room temperature. The pH related to optimal activity of the enzyme was ensured at 25°C in the 100 mM GTA buffer using pH range of 4.0–11.0. Information of the pH stability was obtained by incubating the enzyme with 100 mM GTA buffer (pH 4.0–11.0) for 16 h at 4°C, and residual activity was determined at room temperature (25°C). The analyses related to the optimal activity and the pH stability under various pH conditions were conducted simultaneously with the control test. Four mixtures containing 100 mM GTA buffer (pH 4.0–11.0, at intervals of 1.0 pH unit) and EstCS1 were designated control, reaction 1, reaction 2, and reaction 3, respectively. After reaction under the same conditions with 100 μl 2 N NaOH, the absorbance at 405 nm was measured for quantifying *p*-nitrophenol released from the blended substrate (*p*-nitrophenyl butyrate) in the mixture using an Eon spectrophotometer (BioTek). After that, the final value was derived by comparing the mean of the measurements of the one to three reactions with the control figure to take into account the automatic-hydrolyzation of the substrate.

### Inhibitory Effect of Metal Ions, Detergents, Inhibitors, and Organic Solvents on the Activity of EstCS1

To measure the effect of additives on the activity of EstCS1, various detergents and metal ions were used for incubation with purified and concentrated enzyme in the 100 mM GTA buffer (pH 9.0) at 30°C for 1 h. Following incubation, the remaining activity was gauged under standard assay conditions. The inhibitory effect of several materials (1, 5, and 10 mM concentrations) such as phenylmethylsulfonyl fluoride, dithiothreitol (DTT), ethylenediaminetetraacetic acid, and 2-mercaptoethanol was investigated after incubation with EstCS1 at 30°C for 1 h. The stability test of EstCS1 against organic solvents was performed with 10, 20, 30, and 50% (w/v) of acetonitrile, methanol, dimethyl sulfoxide (DMSO), ethanol, acetone, 2-propanol, and *N*,*N*-dimethylformamide (DMF) in the same condition as it was used for the analysis of inhibitor.

### 3D Structural Prediction of EstCS1

To predict a more reliable structure of EstCS1, two “web-based protein structure prediction systems,” SWISS-MODEL^1^ and I-TASSER^2^, were used. In the case of the SWISS-MODEL system, 50 reference enzymes with structures registered in the protein data bank (PDB)^3^ by the identity score were sorted, and then, tertiary structures of EstCS1 were built. The information of the best model including sequences, distances, and *xyz* coordinates of amino acids in EstCS1 was saved in PDB file format. In addition, multiple alignment among top 5 reference templates with EstCS1 through ESPript 3.0^4^ was performed. Then, the AntheProt 3D viewer (version 1.4.2) to visualize the pdb file in the ribbon and the alpha trace form was used. The catalytic triad and the oxyanion hole of EstCS1 were marked on the 3D figure using the information of the conserved motif (HGGG and GXSXG motif) from results of the multiple alignment. The 3D structure of EstCS1 through the I-TASSER online server was identified. This system mainly depends on the two methods, the “LOMETS,” which identifies a template-based structural simulation by multiple threading approach based on the PDB, and the “BioLiP,” which implements a 3D model through protein function database.

### Hydrolysis of Tertiary Alcohols

For the hydrolysis of tertiary alcohol esters, 100 μg of EstCS1 was gently blended with the substrate–mixture solutions, phenol red (2 mg/ml), and each 25 mM of linalyl acetate, *t*-butyl acetate, and α-terpinyl acetate in the buffering system based on 20 mM Tris–HCl (pH 8.0) (Ngo et al., 2014). The change in color with pH shift was recognized after 5, 10, 30, 60, and 120 min.

### Nucleotide Sequence Accession Number

The nucleotide sequence data of the novel lipolytic gene (*estCS1*) has been deposited in GenBank (accession number. AEQ63714).

## Results

### Screening and Subcloning of the Putative Esterase Gene

**FIGURE 1:**
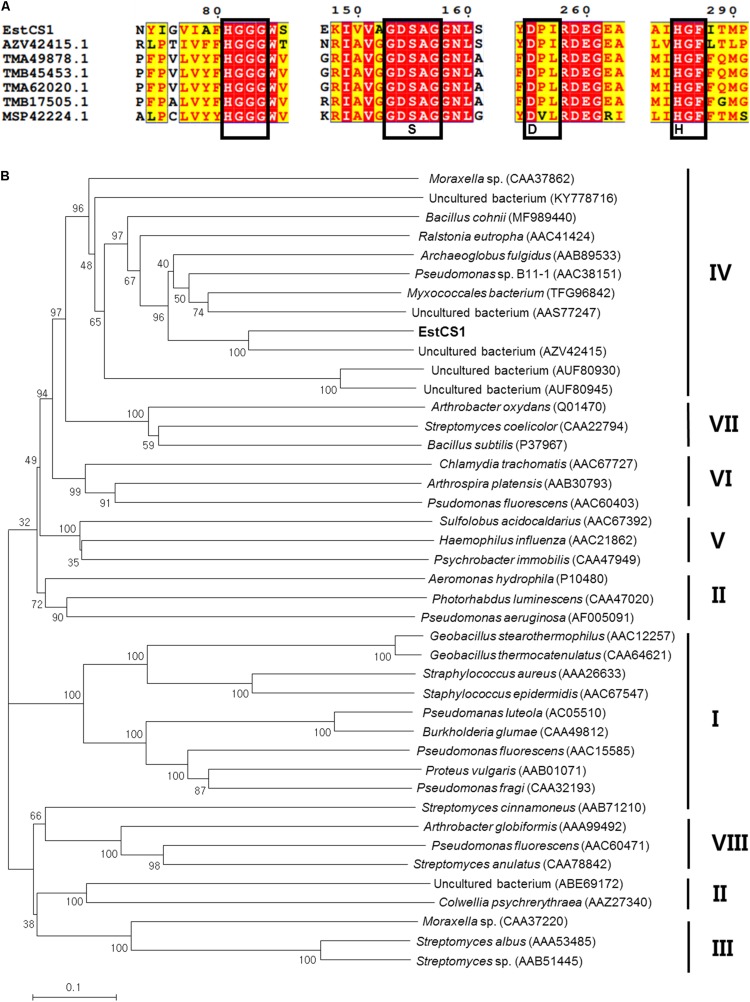
**(A)** Multiple amino acid sequence alignments of EstCS1 and its homologs. AEM45131.1, hypothetical protein (uncultured organism); AAS77247, esterase lipase (uncultured bacterium); ADR31550, EST1 (uncultured bacterium); ABQ11269, lipase esterase (uncultured bacterium); and ABQ11269, lipase esterase (uncultured bacterium). **(B)** Phylogenetic tree based on amino acid sequence of EstCS1 and closely related proteins. The phylogenetic tree illustrates a neighbor-joining method using MEGA4. The numbers at the node indicate bootstrap percentages of 1,000 replicates. The units at the bottom of the tree indicate the number of substitution events. Except for EstCS1, the protein sequences for previously identified families of bacterial lipolytic enzymes were retrieved from GenBank (http://www.ncbi.nlm.nih.gov).

A metagenomic fosmid library constructed from a compost sample was made to screen clones indicating lipolytic activity with a halo formation around *E. coli* colonies on LB–tributyrin agar plate. Primarily, 11 lipolytic active clones among ∼13,000 recombinant fosmid clones were obtained, and the one showing the highest positive activity was selected for further studies of characterization. Subcloning of the gene encoding lipolytic activity within the fosmid was performed with pUC19 and DH5α followed by the digestion of DNA fragments by *Sau*3AI. After selecting subclones with lipolytic activity, sequence analysis of insert DNA within them was performed and the existence of an open reading frame (designated *estCS1*) composed of 927 bp that could be translated into a protein of 309 amino acids with a predicted molecular weight of 34 kDa was confirmed. A BLAST search for sequence comparison among related proteins conducted at the NCBI obviously revealed that the amino acid sequence of EstCS1 had the high identities to lipolytic enzymes from various cultured and uncultured bacteria, including esterase and alpha/beta hydrolases from five cultured bacteria (GenBank accession nos. WP 119116380.1, AZV42415.1, WP 067408583.1, WP 067408583.1, and TFG96842.1; identities of 44–59%), lipases/esterases from two uncultured bacterium (GenBank accession no. AAS77247.1 and ABQ11271.1; identity of 43–46%), and a hypothetical protein from one uncultured bacterium (GenBank accession no. AEM45131.1; identity of 45%). The results of the multiple sequence alignment of EstCS1 with closely related enzymes revealed that there were Ser155–Asp255–His285 residues as the conserved sequence motif of EstCS1. In addition, the catalytic nucleophile serine is at position 155 in the consensus GXSXG pentapeptide and the HGGG motif in the oxyanion hole upstream of the pentapeptide motif; these are characteristics of family IV (HSL family) lipolytic enzymes (Figure 1A; Arpigny and Jaeger, 1999; Kim et al., 2015; Huo et al., 2018; Jayanath et al., 2018; Noby et al., 2018). Phylogenetic analysis using amino acid sequences of EstCS1 and other lipases/esterases also revealed that EstCS1 fell within the cluster including family IV lipolytic enzymes (Figure 1B).

### Overexpression and Purification of EstCS1

The active form of the encoded EstCS1 was overexpressed in the soluble fraction of *E. coli* cells through isopropyl-D-thiogalactopyranoside induction. The yield of 69% from the soluble fraction was acquired through the purification using a Ni–NTA resin, and then, there has been approximately fourfold increase in the specific activity. After denaturing EstCS1, the result of SDS polyacrylamide gel electrophoresis analysis indicated that the enzyme had a molecular mass of nearly 34 kDa. The lipolytic activity of the enzyme was visualized as an apparent band corresponding to the native protein having a size of 34 kDa by a zymogram including an indicator (data not shown).

### Biochemical Characterization of EstCS1

**FIGURE 2:**
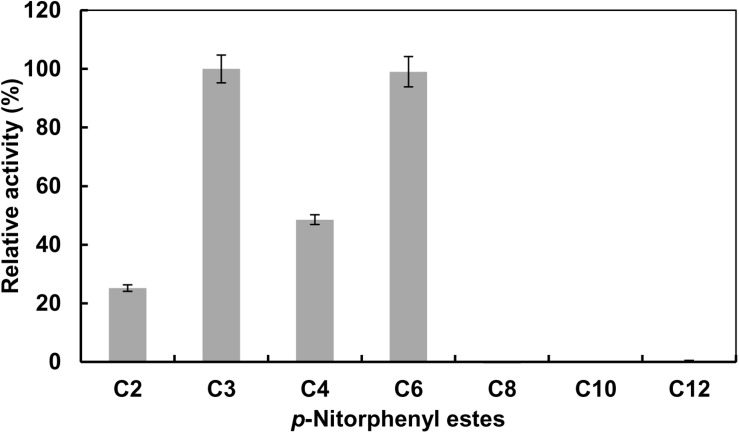
Substrate specificity of EstCS1 toward various *p*-nitrophenyl esters. Substrates used were *p*-nitrophenyl acetate (C2), *p*-nitrophenyl propionate (C3), *p*-nitrophenyl butyrate (C4), *p*-nitrophenyl caproate (C6), *p*-nitrophenyl caprylate (C8), *p*-nitrophenyl caprate (C10), and *p*-nitrophenyl laurate (C12). The error bars represent mean ± SD (*n* = 3).

Substrate specificity of *EstCS1* toward various lengths of acyl chain was investigated by utilizing pNPEs (C2–C12) as substrates. The esterase EstCS1 demonstrated the highest substrate specificity toward *p*-nitrophenyl propionate (C3) and *p*-nitrophenyl caproate (C6) among pNPEs examined. On the other hand, the hydrolytic activity of EstCS1 toward pNPEs with long chains more than C8 was almost not shown (Figure 2). These results implied that the pNPEs with short-chain acyl groups are good substrates for EstCS1, which is in contrast with the pNPEs having acyl groups longer than C8 (Figure 2).

**FIGURE 3:**
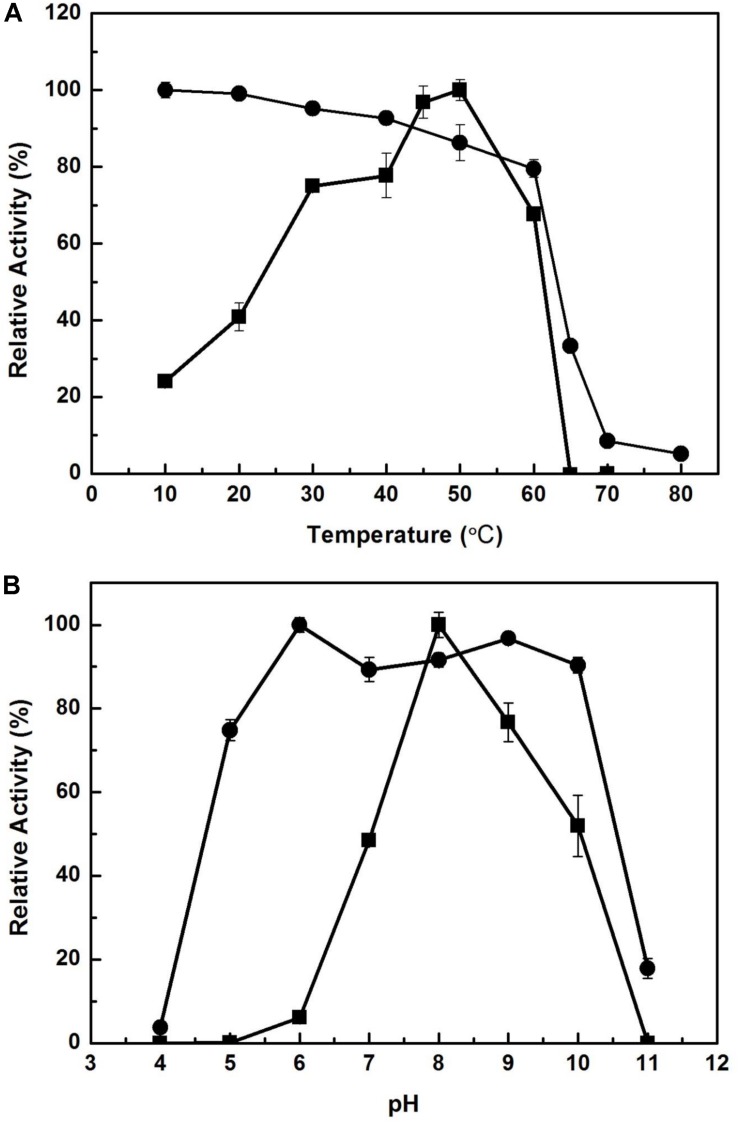
Effect of temperature and pH on the activity of EstCS1. **(A)** The enzyme activity was measured at each temperature under standard assay conditions (■). The enzyme was preincubated at the indicated temperatures, and the remaining activity was determined (∙). **(B)** The enzyme activity was measured at each pH under standard assay conditions (■). The enzyme was preincubated at the indicated pH, and the remaining activity was determined (∙).

The effect of temperature on the activity of EstCS1 was examined over a range of 10–80°C, and the enzyme was clarified to have maximal activity at 50°C (Figure 3A). The EstCS1 was lasting its activity up to 60°C, and its residual activity decreased dramatically in temperatures above 60°C (Figure 3A). In terms of the pH, EstCS1 indicated the optimal activity at pH 8.0 and was stable within the pH range between 5 and 10 (Figure 3B).

### Effect of Additives on the Activity of EstCS1

The activity of EstCS1 dwindled as the level of the concentration of Tweens 20, 40, 60, and 80, Na-deoxycholate, 3-[(3-cholamidopropyl) dimethylaminonio]-1-propanesulphonate, Na-taurocholate, and Triton ×−100 increased, and it was completely inhibited under the presence of SDS (Supplementary Table S1). Ca^2+^, Mg^2+^, and Mn^2+^ ions slightly improved the activity of EstCS1, while EstCS1 activity was restrained by the presence of Cu^2+^ and Zn^2+^ ions (Supplementary Table S2). To investigate the inhibitory effect on the amino acid residues involved in catalysis, various chemical modifiers were applied to EstCS1. The activity of EstCS1 declined dramatically by phenylmethylsulfonylfluoride, as shown in lipolytic enzymes containing a serine residue in the conserved pentapeptide GXSXG of their active site (Supplementary Table S2; Soror et al., 2007; Jayanath et al., 2018). In the condition with 1–10 mM ethylenediaminetetraacetic acid, the activity of EstCS1 was maintained, which suggested that metal cofactors are not essential for the activity of EstCS1 (Supplementary Table S2). DTT, a strong reducing agent, is generally used to eliminate disulfide bonds within proteins and to hinder cysteine residues of proteins from forming intramolecular and intermolecular disulfide bonds. In the case of EstCS1, the presence of 1 and 5 mM DTT increased its activity, indicating that thiol groups may be crucial for enzyme activity (Côté and Shareck, 2010; Peng et al., 2014). The enzyme activity of EstCS1 was sustained in 1 and 5 mM of 2-mercaptoethanol, but its activity was inhibited strongly when 10 mM of 2-mercaptoethanol was added (Supplementary Table S2).

### The Effect of Organic Solvents on the Activity of EstCS1

To figure out the effect of solvents on activity of EstCS1, various organic solvents were used at final concentrations of 10, 20, 30, and 50% (v/v). In the presence of ethanol, 2-propanol, acetonitrile, and acetone from 10% (v/v) to 30% (v/v), the activity was increased or retained. Whereas enzymatic activity slightly decreased when methanol and DMF from 10% (v/v) to 30% (v/v) were added (Table 1). The addition of 50% (v/v) acetone slightly decreased enzymatic activity, too. However, the addition of 50% (v/v) 2-propanol sharply decreased enzymatic activity (Table 1). Other than those, incubation for 1 h with 50% (v/v) methanol, acetonitrile, ethanol, DMSO, or DMF showed the enzymatic activity in the range of 43.0–66.1% (Table 1).

**TABLE 1:** Effects of organic solvents on the EstC1.

**Solvent**	**Remaining activity (%) at various solvent**			
	**concentrations (%) of**			
	**10**	**20**	**30**	**50**
Control	100	100	100	100
Methanol	95.0	92.7	90.8	43.4
Ethanol	97.8	99.9	101.4	50.3
2-Propanol	119.0	111.2	115.8	25.2
Acetonitrile	95.1	110.5	101.1	43.0
DMSO	91.8	86.0	74.4	66.1
Acetone	109.4	103.9	100.0	81.8
DMF	91.6	96.8	87.4	51.2

### Prediction of 3D Structure, Active Site, and Oxyanion Hole of EstCS1

**FIGURE 4:**
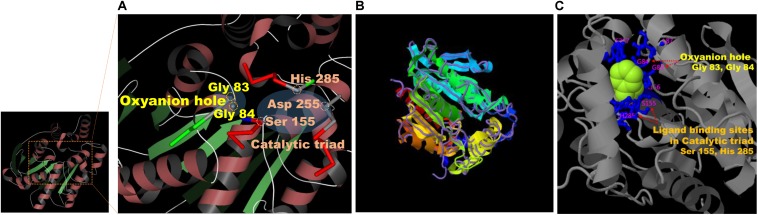
**(A)** Model 2 structure and major residues based on the analysis of the SWISS-MODEL system (https://swissmodel.expasy.org). **(B)** The structural prediction of the model 3 (ribbon parameters) with the structurally most analogous protein (4YPV, purple line) in PDB using the I-TASSER system (https://zhanglab.ccmb.med.umich.edu/I-TASSER/). **(C)** Visualization of the combination between a phenylmethanesulfonic acid (PMS) and the major residues predicted to be important for ligand biding and activity of model 3 from the multiple function annotation analysis of the I-TASSER.

Results on the web-based prediction of 3D structure using the SWISS-MODEL system showed that 50 reference enzymes with structures registered in the PDB (see text footnote 3) are related to the sequence information of EstCS1. Among them, the best two predicted models in terms of the identity, coverage, global model quality estimate (GMQE), and qualitative model energy analysis (QMEAN) were derived from 3ZWQ (model 1, sequence identity, 46.88%; coverage, 0.93; GMQE, 0.73; QMEAN, −2.97) and 4YPV (model 2, sequence identity, 43.75; coverage, 0.93; GMQE, 0.73; QMEAN, −1.91) in the PDB. The model 2 based on the structure of the 4YPV was assumed to be a monomer, but the predicted structure from 3ZWQ was estimated to be a homo-tetramer. Combining these results with the molecular mass of EstCS1 suggested that model 2 has better predictive structure. In addition, the sequence and position of the conserved motif and the active site of EstCS1 from alignment results of the T-COFFEE and the ESPript 3.0 were identified and then integrated them with model 2 into 3D images using the AntheProt 3D viewer (Figure 4A).

Based on the I-TASSER using different mechanisms for reliable reasoning, model 3 derived from top 4 proteins including 4YPV, 6KMOA, 5JD4, and 1JJI (*Z* score: 3.60, 4.36, 0.88, and 1.21, respectively), and its *C* score was 1.53. The model 3 was estimated to be a monomer, and it showed the highest TM score in structure with 4YPV through the TM-align structural alignment program (Figure 4B). The prediction of ligand-binding sites showed the highest *C* score of 0.82 when 3H17A data were applied. Besides, when summarizing the top 5 predicted results of ligand-binding sites and active sites, the most importantly anticipated residues were Gly 83 and Gly 84 in the HGGG motif, Ser 155 in the GXSXG motif, Asp 255, and His 285 residues (Figure 4C).

### Hydrolysis of Tertiary Alcohol Esters by EstCS1

The ability of EstCS1 to hydrolyze esters of tertiary alcohols was studied using linalyl acetate, *t*-butyl acetate, and α-terpinyl acetate as substrates and by pH indicator-based colorimetric assay on the release of acetic acid. The results showed that EstCS1 weakly hydrolyzed *t*-butyl acetate and linalyl acetate after incubation for 120 min (Supplementary Figure S1).

## Discussion

The metagenomic library of the compost sample was investigated for screening lipolytic enzymes based on the activity-screening method. A lipolytic enzyme, designated EstCS1, was identified in this study. In view of the phylogenetic tree founded on amino acid sequences, the selected EstCS1 was considered as a member of family IV (Figure 1B). The EstCS1 was found to have novel amino acid sequence showing identities <59% to lipolytic enzymes from various cultured and uncultured bacteria. Amino sequence analysis indicated that the EstCS1 contained a putative catalytic triad composed of Ser155–Asp255–His285 and conserved sequence motif of esterase/lipase, GXSXG (Figure 1A; Arpigny and Jaeger, 1999). Two C-terminal conserved motifs, -DPLR- and - HGF-, were also clarified in the EstCS1 as shown in some closely related enzymes (Lee et al., 2004; Hong et al., 2007; Nacke et al., 2011; Jiang et al., 2012; Ko et al., 2012; Noby et al., 2018).

EstCS1 had a preference toward shot-chain *p*-nitrophenyl esters up to C6 as substrates (Figure 2). EstCS1 indicated optimal activity at 50°C, and its activity was stably sustained in the temperature range of 10–60°C (Figure 3A). According to the thermostability data, EstCs1 was shown to be a moderately thermophilic or thermostable esterase, which has been described in some metagenomic esterases belonging to family IV (Rhee et al., 2005; Choi et al., 2013; Noby et al., 2018). The thermostability of EstCS1 may have originated from a moderately thermophilic or thermostable microorganism mainly existing in compost maintaining high temperatures during the composting process. The activity of EstCS1, meanwhile, was measured at various pH and indicated optimal activity at pH 8.0. In addition, over 80% activity of the enzyme was sustained from pH 6.0 to 10 after incubation for 1 h (Figure 3B). Given the results so far, EstCS1 is considered as a moderately thermophilic and alkaliphilic esterase (Figure 3).

Biocatalysis are especially advantageous when they improve the substrate solubility in organic solvents and suppress undesired side reactions (Alex et al., 2014; Jayanath et al., 2018). In general, the stability of enzymes in hydrophilic organic solvents is diminished because of the solvent penetration effect deforming the enzyme surface (Yang et al., 2004; Park et al., 2013). The stability of esterases affected by organic solvents is advantageous when they are utilized to biotechnology and industrial fields (Kawata and Ogino, 2010; Jayanath et al., 2018). As proposed in the numerous protein engineering approaches, a directed evolutionary approach was adopted to acquire stable enzymes in hydrophilic organic solvents (Park et al., 2013). In this study, it was clearly revealed that EstCS1 has high stability in polar organic solvents (Table 1). When exposed to methanol, ethanol, acetonitrile, 2-propanol, acetonitrile, DMSO, acetone, and DMF of 30% (v/v), the enzyme was shown to retain its activity to over 70% (Table 1). Besides, EstCS1 turned out to possess an activity of more than 50% in 50% ethanol, DMSO, acetone, and DMF (v/v) (Table 1). To our knowledge, there are few studies on enzymes having stability like this in condition with high solvent concentrations of 30–50% (v/v) among characterized lipolytic enzymes categorized into the family IV (Jiang et al., 2012; Ko et al., 2012; Oh et al., 2012; Fang et al., 2015; Kim et al., 2015; Huo et al., 2018; Jayanath et al., 2018).

The ability of EstCS1 to cleave sterically hindered esters between two tertiary alcohols is notable. Given the results of the multiple alignment and structure modeling, EstCS1 shared a GGG(A)X motif in the oxyanion hole close to the active site as shown in most esterases and lipases with the activity at the sterically hindered tertiary alcohols (Henke et al., 2002; Kourist et al., 2007; Jin et al., 2012; Huo et al., 2018; Jayanath et al., 2018). In particular, the residues belonging to the oxyanion hole had been estimated as Gly 83 and Gly 84 in the GGG(A)X motif. They have NH groups forming hydrogen bond with the nucleophilic oxygen of a substrate. The first tetrahedral intermediate of total EstCS1–substrate reaction was created through this chemical combination, which stabilized the negative charge of the transition state. The sequential exchange of an electron among those in the oxyanion hole and the residues including Ser155, Asp255, and His285 in the active site lowered the activation energy necessary for the reaction, which finally push the substrate to the carboxylic acid. Enlarging the active site surrounding the oxyanion hole thereby providing an enlarged substrate binding site was considered as a reason for activity related to GGG(A)X (Henke et al., 2002; Kourist et al., 2007; Huo et al., 2018).

EstCS1 identified from this study was characterized to be a moderately thermophilic esterase that can cleave esters between two tertiary alcohols and has a high stability against organic solvents. Those properties of EstCS1 are worthy of a lot of attention in various biotechnological applications associated with organic solvents, for example, ester synthesis and transesterification reactions. This study also suggests that the metagenomic approach is still worthy of use to identify novel enzymes with high potential for industrial applications and to expand our perspective of biodiversity for screening novel enzymes.

## Data Availability Statement

The datasets generated for this study can be found in the GenBank/Accession number AEQ63714.

## Author Contributions

J-MP performed the experiments of this study and reviewed the manuscript. C-HK performed the experiments of this study. S-MW supported some of the experiments of this study. K-HO discussed the results of this study. J-HY designed this study and wrote the manuscript.

## Conflict of Interest

The authors declare that the research was conducted in the absence of any commercial or financial relationships that could be construed as a potential conflict of interest.

## Footnotes

1 https://swissmodel.expasy.org

2 https://zhanglab.ccmb.med.umich.edu/I-TASSER/

3 rcsb.org

4 http://espript.ibcp.fr/ESPript/ESPript/index.php
