# Supplementary material for: Characterization of a Novel Moderately Thermophilic Solvent-Tolerant Esterase Isolated From a Compost Metagenome Library
Source: Front Microbiol. 2020 Jan 24;10:3069. doi: 10.3389/fmicb.2019.03069 (PMC6993047; doi:10.3389/fmicb.2019.03069)
Supplement: Supplementary file 1 [file Data_Sheet_1.pdf]

frontiers

Supplementary Material

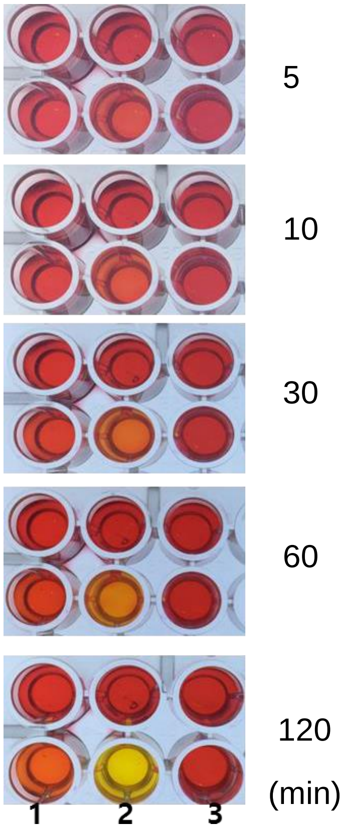

SUPPLEMENTARY FIGURE S1 Tertiary alcohol ester hydrolysis assay. The hydrolysis of tertiary alcohol esters (TAEs) by purified enzymes was measured using a pH-shift assay. Lane 1, linalyl acetate; Lane 2, *t*-butyl acetate; Lane 3, α-terpinyl acetate. The reaction times were 5, 10, 30, 60 and 120 min from top to bottom. At each stage, the first and second rows included buffer and enzymes, respectively.

SUPPLEMENTARY TABLE S1 Effect of detergents on the EstC1

Compounds	Relative activity (%)* at concentration (%, v/v) of		
	0.1	1	5
Tween 20	75	20	9
Tween 40	71	29	4
Tween 60	66	27	3
Tween 80	52	24	12
Triton X-100	73	31	7
Na-taurocholate	62	35	8
Na-deoxycholate	75	30	15
CHAPS	74	58	23
SDS	0.4	0	0

*The relative activities are given as a percentage of the activity in the absence of detergents

SUPPLEMENTARY TABLE S2 Effect of metal ions and inhibitors on the EstC1

Compounds	Relative activity (%)* at concentration (mM) of		
	1	5	10
$CaCl_2$	129	113	126
$CoCl_2$	112	87	23
$CuSO_4$	25	6	6
$MgSO_4$	115	121	102
$MnSO_4$	131	113	105
$FeSO_4$	110	50	31
$ZnSO_4$	23	14	12
EDTA	103	102	84
PMSF	5	5	7
DTT	124	116	17
2-Mercaptoethanol	105	100	3

*The relative activities are given as a percentage of the activity in the absence of cations, EDTA, PMSF, DTT and 2-mercaptoethanol
